# Diseases of the Eye

**Published:** 1855-10

**Authors:** George Critchett


Diseases of the Eye.—The treatment of strumous ophthalmia naturally divides itself into constitutional and local, which may be dwelt upon separately, for the sake of arrangement, though at the same time it must be born in mind that they bear an intimate relation to each other; for, while it must be admitted that all these cases are in some measure due to constitutional causes the local disease often persists long after the original and exciting systemic cause has passed away. There are three principal digressions from the normal standard that I have particularly noticed as favorable for the incubation of this form of ophthalmia. The first of these I would designate the 'inflammatory type.' It is rapidly developed, the local symptoms are acute, there is severe pain in the eyes, attended with profuse lachrymation of a scalding character, red, swollen lids, and spasmodic closure. The constitutional symptoms exhibit a hot and dry skin, a rapid pulse, foul tongue, loss of appetite, arrest of all the natural secretions, constant restlessness. The treatment, under these circumstances, consists chiefly of salines, antimonials, mercurial purgatives, and the use of the warm bath: this latter is peculiarly useful in such cases. The diet must be light, and not either stimulating or too nutritious. At the same time care is required not to persevere in this plan too long, not in fact a day longer than the state of the secretions indicate its propriety, otherwise a feeble condition is induced, and the disease continues in a subacute or chronic state. Much discrimination is needed in making out these shades of difference, and success in the treatment mainly depends upon our doing so.

The second class of cases that seem to deserve special notice may be denominated the 'irritable type.' In these cases the mucous membranes are congested and disordered, and their secretions increased in quantity and vitiated in quality; the tongue and lips are aphthous and swollen; the nose excoriated; the skin inflamed: combined with this there is often a tendency to prolapse of the rectum and chronic diarrhœa; this condition frequently follows one or other of the exanthemata, and may be kept up by the presence of worms. You may readily recognize this form of disease by the general outpouring from the mucous outlets. Unsuitable food and want of cleanliness are fertile sources of this deranged state of the mucous membranes.

In the treatment of these cases all causes of irritation must be removed; if worms are suspected, one or two drastic aperients may be required. The secretions may be improved by a mild mercurial and an alkali. The diet must be carefully attended to; many of these symptoms result from crude, ill-digested food, improper in quality or superabundant in quantity. The children of the poor are very much mismanaged in this respect. At the Ophthalmic Hospital, I always gave a laconic, but, as I believe, an effective formula—"stale bread-and-butter, milk-and-water and a little meat once a day." It often happens, however, that after all these means have been carefully employed, the irritation of the mucous membrane remains, which purgatives only seem to increase. Under these circumstances I found the utmost possible benefit from Battley's solution, regulating the dose by the age of the patient, allowing a minim for each year, and giving it once or twice in the twenty-four hours. It seems, in these cases, to act like a charm, removing the diarrhœa, checking the other morbid secretions, improving the appetite, allaying restlessness, and subduing the most urgent and distressing symptoms, namely, the extreme photophobia. Much prejudice exist in the minds of many against the use of opium in any form in children; but extensive experience has convinced me of the great value of this remedy in suitable cases. Warm baths soothe and allay irritation. Tonics of any kind are not well borne, and seem to aggravate all the symptoms.

The third division of these cases may be termed the 'asthenic type.' It is found most commonly among the children of our poorer population. The chief symptoms are emaciation and pallor, with a rapid and weak pulse, cold extremities, moist tongue, frequent perspirations, and are quite compatible with, and coexist with a considerable amount of ophthalmia and extreme photophobia. It is in this group of cases that we commonly find the intermittent form of the disease. Towards evening the child will open its eyes, and play about as usual; but the symptoms will recur with the same intensity the next morning. I have observed that a tonic treatment is most valuable in these cases; they often come before our notice at the Ophthalmic Hospital. We find such patients suffering from the effect of bad ventilation and insufficient and unwholesome food; and sometimes, in addition to this, active antiphlogistic treatment may have been employed, with a view of subduing local inflammation. It is remarkable how rapid a change is produced by the adoption of every available means for developing power in the system. Where intermittance of symptoms and periodicity can be traced, quinine seems to act like a specific; the dose must be, of course, regulated by the age of the patient, but rather a large dose can be borne, and seems to be required. Where the fibre is very lax, and the limbs feel flabby, steel is useful, and seems to be indicated, and the two may sometimes be combined with great advantage. The combined salt of iron and quinine is a convenient form for its exhibition. At the same time a liberal diet, with a moderate amount of stimulus, must be given; and fresh air, and, if possible, sea air and all such means as are calculated to develope power.

Many medical men, judging by the severity of the local symptoms, and losing sight of the general debility of the system, treat such cases antiphlogistically, limiting the patient to spoon-diet, and acting upon the various secretions. When a case so treated presents itself suffering from all the adverse circumstances which a feeble state of system has induced, it is often remarkable to observe the rapid improvement that takes place under an entire change of management. Tonics and a liberal diet may be given altogether regardless of the severity of the local symptoms and of the intensity of the intolerance of light. If this plan can be fully carried out, its favorable effect is speedily observed; but our chief difficulty consists in counteracting the enfeebling influences by which such cases are surrounded, such as impure air, unwholesome and insufficient food, etc. When the strumous diathesis is very marked, the glands enlarged, upper lip thickened, cod-liver oil may be given with advantage, either alone or combined with steel. If the tongue is foul or the secretions disordered, it is a good plan to commence the treatment with an emetic, and then to exhibit tonics. Where the secretions are very abundant, opiates may be combined with tonics very advantageously. Where this asthenic condition is complicated with much mucus and cutaneous irritation, such as I have before described, with swollen nose and lips, enlargement of the glands, acrid secretions, and a cold, pasty, flabby surface, I have observed remarkable improvement in all these symptoms, from the exhibition of arsenic, in doses proportioned to the age of the patients. About two minims of the liquor arsenicalis is the dose I generally give, three times a day, immediately after meals. The effect must be carefully watched, and the medicine must not be given at any one time for a longer period than a fortnight or three weeks. The beneficial effect is often rapid and striking, and it may be given in a state of system for which no other medicine seems to be quite suited. As regards the use of mercury in these cases, I believe that in by far the greater majority it is very injurious; but instances do occur, though they are rare and difficult to detect, in which this medicine is useful. I can only indicate them as belonging to the class of strumo syphilitic, in which some syphilitic taint is engrafted upon and combined with struma and produces a train of symptoms in many respects resembling some forms of tertiary syphilis. I have met with cases so closely resembling the more remote effects of a primary sore, that if it were not for the age of the patient, you might suppose it was the result of the direct influence of this poison. I am not now speaking of those cases that occur soon after birth, and are manifestly syphilitic, but rather of such as appear at ages ranging from about seven to twelve; the cornea and iris and even the deeper textures are often involved, and the intolerence of light is very extreme. Minute doses of mercury, as for instance one or two grains of the gray powder, or one ninetieth of a grain of the bichloride, twice or three times a day, seem to act very beneficially both upon the general health and the local symptoms. I must, however, again urge the extreme importance of correctly diagnosing this peculiar complication, otherwise more harm than good will be done by the exhibition of this medicine.

Before I leave the subject of constitutional treatment, I must not omit to mention that most efficient of all means for restoring strength in a strumous diathesis—I mean change of air, particularly to the sea—this expedient will sometimes effect a cure when all else has failed. Not only is it useful in removing present mischief, but it often prevents a relapse, a strong tendency to which constitutes one of the most distressing features of such cases.

We may now pass on to the consideration of the local treatment of strumous ophthalmia, a point second only in importance to the constitutional, since we find a judicious combination of topical means with general treatment essential to success.

Local treatment may be arranged conveniently under the following heads: Local depletion; Counter-irritation; Local stimulation to the lids and to the eye; Local sedatives. In the earlier stages of an attack, where the symptoms are acute, the vessels full and numerous, and of bright-red color, or in a sudden relapse of an old standing case, in which you can trace fresh organized deposite or a false membrane on the cornea, the abstraction of blood, by means of leeches, is often useful; they may be repeated, with advantage, once or twice, but I have usually found it useless to continue this plan for any time; all the benefit likely to accrue from it is very speedily obtained; a prolongation of local depletion is apt to enfeeble the system, without any corresponding subsidence of the local disease. In less acute cases, where the vascularity is not so prominent a symptom, or where it has been diminished by local depletion, blisters are very useful; they must be frequently repeated, and, in mild cases, will effect complete relief of the more prominent symptoms. Mr. Tyrrell dwells strongly upon the advantage of this form of temporary counter-irritation in relieving intolerance of light, and seems to rely upon it almost exclusively as a local means in these cases. My own experience does not allow me fully to subscribe to this view. In the more severe cases, blisters fail; and in some forms of the disease, in which mucous and cutaneous irritation coexist, they seem to be decidedly contra-indicated. A modification of this plan, though possibly acting in a very different way, has recently been strongly advocated. It consists in applying some powerful local stimulant to the eyelids. Some use for this purpose the solid nitrate of silver, moistened, and brushed lightly over the lids; others paint the tincture of iodine on the lids. Whichever is selected, care must be taken that it does not find its way into the eye; and it usually requires to be repeated every four-and-twenty hours, for a few days, till a decided impression is made on the skin. The advocates of this plan attribute its efficacy to the direct influence of the stimulus upon the extreme branches of the fifth pair of nerves: upon an abnormal irritability of which they believe the photophobia to depend. Whether the explanation be correct or not, I have no doubt of the advantage of the plan in some cases. I have tried it very extensively, and have observed that where it is useful its effect is very rapid and complete, but that it is only in a certain limited number of cases; in many others, it has had no appreciable effect. The important and difficult point to ascertain is, some guide or indication to its applicability—whether the use of this method must be tentative and empirical, or whether we can select cases in which a favorable result may be predicated. It appears to me that this method is peculiarly applicable to those cases in which vascularity and other evidence of diseased action are slight, and photophobia exists as the prominent and principal symptom, and in which the disease is not of very long standing. I generally use the tincture of iodine in preference to the nitrate of silver, because it occasions less pain, is less liable to produce vesication, and is equally efficacious. With regard to the employment of solutions of the nitrate of silver, and other local stimuli, to the conjunctival surface, in strumous ophthalmia, I find them, in a majority of cases, decidedly injurious, and, under these circumstances, they seem very much to retard the cure, and to exert a baneful influence for some time after they have been discontinued. The presence of photophobia, however, does not alter the rules I have already laid down for your guidance, in a former lecture, with regard to the employment of such means. If the secretion is of a puro-mucous character, stimuli will be of service, but they will not subdue the disease single-handed, as in pure catarrhal ophthalmia, but must be combined with other suitable means, both constitutional and local, and if the discharge becomes aqueous in character, the local treatment must be altered. Cases do occasionally occur in which the symptoms are severe and protracted, and in which I have observed, both in my own practice and in that of others, great benefit from the use of nitrate of silver, although none of the conditions above-mentioned, as indicating this remedy, were present. My present motive for its employment, under such circumstances, has been the exhaustion of all other available means, and rather as a *dernier ressort* than as the result of any scientific induction. Such occasional and accidental exceptions to a rule do not at present advance our knowledge of treatment, but ought in candor to be mentioned, and may ultimately lead to the elucidation of a fresh law. In those forms of strumous ophthalmia, in which irritability is a prominent symptom, in which the disease is limited to the conjunctiva, forming aphthæ and phlyctenulæ, attended with aqueous secretion of a frothy character, local sedatives are very useful. Of these, perhaps the best is the opium wine of the Pharmacopœia, dropped into the eye two or three times a day; it generally causes rather severe pain, that lasts several minutes, but is followed by a marked improvement in the symptoms. Prussic acid is highly extolled by some authorities; it may be used in the form of vapor, or as a lotion, or drops, diluted in the proportion of a drachm of prussic acid, of the Pharmacopœia strength, to an ounce of distilled water. I have certainly observed great advantage from its employment in several cases. I can speak with more confidence of the opium wine than of any other form of sedative as a local application; it is an old but very valuable remedy that has fallen into unmerited neglect. This has probably arisen from its too indiscriminate use, and from a want of care and judgment in selecting cases suitable for its employment.

It will be seen in the foregoing remarks that I have endeavored to map out some of the more salient phases under which this very complicated disease presents itself to our notice, and to indicate the treatment best suited for each form: but I feel, in common with all who have an opportunity of extensive observation, how extremely difficult it is to convey in a lecture an accurate idea of these distinctions, even when well marked, and how impossible it is by such an imperfect medium to indicate the various shades of difference that are constantly presenting themselves to our notice, and the slight gradations by which one form glides into and combines with another, necessitating constant modifications of treatment, and presenting many difficulties even to the most experienced. At the same time I am not without hope that the distinctions I have endeavored to draw may be found of practical value as starting-points for individual observation—may prevent any flagrant error in treatment, and may explain how it is that so many different and even antagonistic systems have found advocates, and have been productive of good results. After all, it must be admitted that cases sometimes present themselves in which the treatment must be tentative; you must administer such varied means as have been found useful in the succession of their probable efficiency, remembering that many of the worst forms of this disease depend upon a state of constitution over which we possess but little power, and therefore that they must be necessarily protracted. In conclusion, let me remind you that there is a gradual curative tendency in all such cases, and hence much care is needed not to thwart these spontaneous efforts by too active or depletory a mode of treatment, the effect of which must be to undermine those powers, and paralyse those efforts, by means of which Nature will gradually effect a cure.—*George Critchett, London Lancet.*

